# Mycelium development in *Streptomyces antibioticus *ATCC11891 occurs in an orderly pattern which determines multiphase growth curves

**DOI:** 10.1186/1471-2180-5-51

**Published:** 2005-09-15

**Authors:** Angel Manteca, Marisol Fernandez, Jesus Sanchez

**Affiliations:** 1 Universidad de Oviedo, Facultad de Medicina, Area de Microbiologia, Departamento de Biologia Funcional, 33006, Julian Claveria s/n, Oviedo, Spain; 2 Laboratorio de Proteomica, Centro Nacional de Biotecnologia, Cantoblanco, 28049 Madrid, Spain

## Abstract

**Background:**

The current model for the developmental cycle of *Streptomyces *confluent cultures on agar surface is based on the assumption that the only differentiation takes place along the transverse axis (bottom-up): a vegetative (substrate) mycelium grows completely live and viable on the surface and inside the agar until it undergoes a death process and differentiates to a reproductive (aerial) mycelium which grows into the air. Hence, this vertical description assumes that the development in the pre-sporulating phases is more or less homogeneous in all zones of the plate surface.

**Results:**

The work presents a detailed analysis of the differentiation cycle in *Streptomyces antibioticus *ATCC11891 considering a different spatial dimension: the longitudinal axes, represented by the plate surface. A previously unsuspected complexity during the substrate mycelial phase was detected. We have demonstrated that the young substrate hyphae suffer an early death round that has not been previously described. Subsequently, the remaining mycelium grows in successive waves which vary according to the density of the spore inoculum. In the presence of dense inocula (1.5 × 10^6 ^spores per plate), the hyphae develop in regular circles, approximately 0.5 cm in diameter. By contrast, with highly diluted inocula (6 × 10^3 ^spores per plate), aerial mycelium develops initially in the form of islands measuring 0.9 mm in diameter. Further mycelial development occurs between the circles or islands until the plate surface is totally covered. This pattern persists throughout the entire developmental cycle including the sporulation phases.

**Conclusion:**

An early death round during the substrate mycelial phase of *Streptomyces antibioticus *ATCC11891 takes place prior to successive growth periods in surface cultures. These developmental periods in turn, determine the shape of the complex multiphase growth curves observed. As shown here, these results also apply to other *Streptomyces *strains and species. Understanding these peculiarities of the *Streptomyces *developmental cycle is essential in order to properly interpret the morphological/biochemical data obtained from solid cultures and will expand the number of potential phenotypes subject to study.

## Background

*Streptomyces *is a naturally occurring bacterium in soil and is likely to be present in aquatic habitats as well [1]. Since the early discovery of this microorganism's ability to to produce clinically useful antibiotics [2,3], the bacterium has received tremendous scientific attention [4]. Furthermore, other noteworthy characteristics, such as its remarkably complex developmental features, make this microorganism an interesting subject of study. Early on, *Streptomyces *was seen to form two distinct structures when grown on culture surfaces [5]: a substrate (vegetative) mycelium and an aerial (reproductive) mycelium. Substrate mycelium, which is assumed to grow into the medium, has a mean diameter of 0.7 μm and is bound by a 0.01–0.02 μm thick mucopeptide cell wall (reviewed in 6). This mycelium is assumed to be present in different stages of cellular degeneration during all growth phases. Early reports stated that aerial hyphae were the result of simple branching of substrate hyphae [7] and were preceded by a short period of decreased macromolecular synthesis [8]. One important feature of the aerial hyphae is that their outer surface is covered with a superficial fibrous sheath [7,9-11]. All these reports described the *Streptomyces *life cycle as a bottom-up (substrate-aerial) process. Consequently, it was assumed that development was uniform throughout the entire plate surface.

Our previous works have presented a detailed analysis of *S. antibioticus *development [12-14]. To obtain a reliable picture of the cell death phenomena that accompany this process, we have used a technique to analyse bacterial viability that involves staining the nucleic acids of the damaged (leaky) cells with propidium iodide (PI) [12]. This dye only enters cells with damaged membranes and substantially enhances fluorescence by binding to nucleic acids with little or no sequence preference (references in 15). PI staining, alone or in combination with fluorescein derivatives, has been widely used for cell death analysis in bacteria [16-19] and also in eukaryotic cells [20]. The reliability of this method has been also assessed in *Streptomyces *in submerged [13,21] and surface [12,14] conditions. Here we have extended our studies to a third dimension: the longitudinal axes on the plate surface. As illustrated below, this perspective is fundamental to understanding the developmental cycle of this bacterium.

## Results

### Confocal laser-scanning fluorescence microscopy (CLSM) analysis of development-linked cell death processes of *Streptomyces antibioticus *ATCC11891 in confluent surface cultures

**Figure 1:**
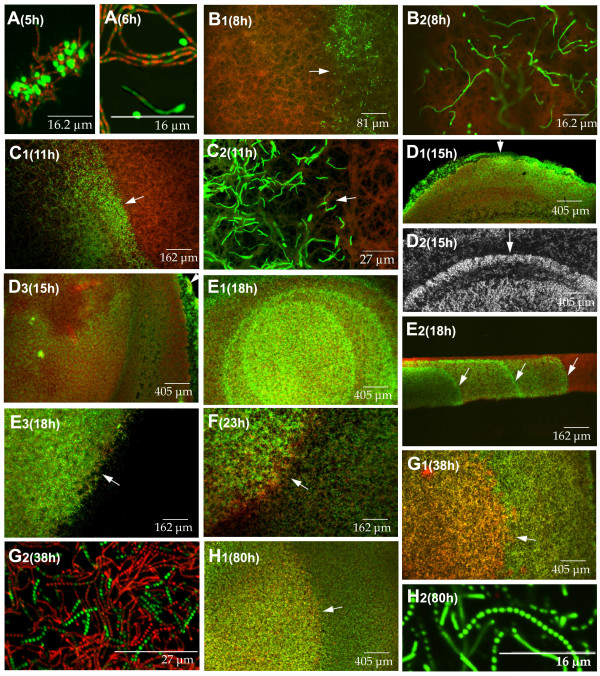
Confocal laser-scanning fluorescence microscopy analysis of the development-linked cell death processes of *Streptomyces antibioticus *ATCC11891 in confluent surface cultures. Developmental phases (A-H) and culture times (hours) are indicated. Picture D_2 _was obtained under the phase contrast microscope. The other images correspond to culture sections stained with SYTO 9 and propidium iodide. E_2 _is a cross section view; the other images correspond to longitudinal sections (see methods). Arrows in E_2 _indicate the eccentric circles of live mycelium developing from the bottom upwards, forming distinct layers with well-defined boundaries. Arrows in the rest of the images indicate circle edges. For details see text.

Figure 1 presents a global perspective of some of the most relevant features of the different developmental steps analysed in *Streptomyces antibioticus *ATCC11891 on surface GAE cultures. To facilitate a sequential view of the process, we have divided it into several phases (A-H; Figure 1).

Phase A (0–7 hours) consists of germination and early hyphae development. When the spores are spread out on the surface (normally with a bent glass stick), all of them are viable (stained green) and remain in relatively large groups, probably owing to their hydrophobic properties (not shown). Germination begins at 4 hours by the asynchronous emission of a single (or less frequently, double) germ tube (Figure 1A, 5 and 6 hours). Most of these young hyphae undergo a very early death process (Figure 1A, 5 and 6 hours), which is remarkably symmetrical in a large proportion of the cases: live and dead segments alternate in a highly regular fashion within the same hyphae (Figure 1A, 6 hours); we have named them variegated hyphae [14]. This death process affects most of the young hyphae, although a small proportion of spores emit a germ tube that, while it is initially totally viable (Figure 1A, 6 hours), will eventually die. This is not unexpected, given that the spores are not distributed homogeneously throughout the plate and the microenvironment encountered by each of them may differ.

In Phase B (7–10 hours), the plate is completely covered with a thin layer of variegated mycelium (Figure 1B_1_, 8 hours). At this stage, the appearance of thin, green rings (Figure 1B_1_, 8 hours) measuring approximately 0.5 cm in diameter is particularly noteworthy. These rings are formed by the hyphae of late-germinating spores that remain live (high magnification in figure 1B_2_, 8 hours), and contrast with the mycelium originating from the previously germinated spores; hence, the variegated appearance. The center of the circle delimitated by the ring, as well as the mycelium located between the rings, is made up of variegated hyphae.

During Phase C (10–14 hours), the young, non-variegated hyphae referred to in Phase B that comprise the border of the circles undergo rapid, profuse growth (Figure 1C, 11 hours).

**Figure 3:**
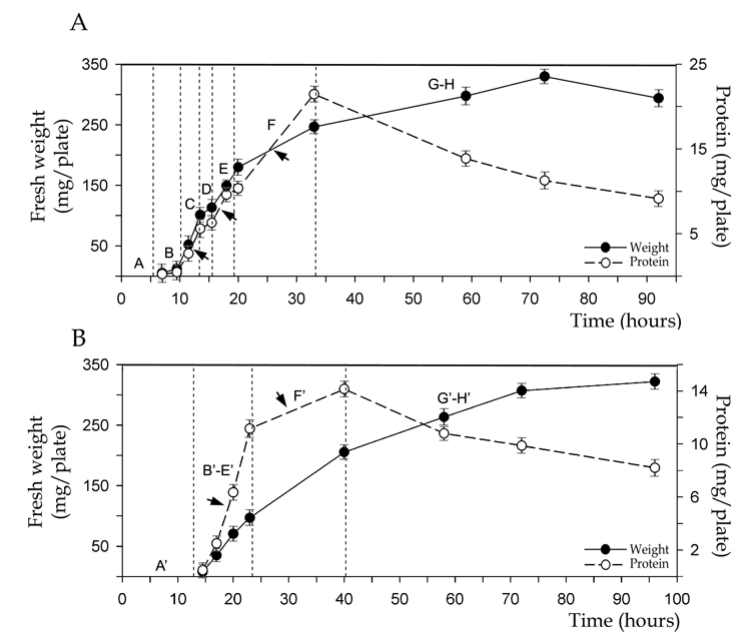
Growth curves (total protein and fresh weight per plate) of *Streptomyces antibioticus *ATCC11891 on surface GAE cultures developed from normal (A, 1.5 × 10^6 ^spores per plate) and diluted (B, 6 × 10^3 ^spores per plate) inocula. Arrows point to the exponential growth stages. Developmental phases (A-H and A'-H') are reflected in the curve. Error bars indicate ± SD.

Phase D (14–16 hours) shows an overall decline in the grow rate (see below, Figure 3A). The borders of the circles cease to grow and live mycelium begins to slowly develop in the center (Figure 1D, 15 hours). This mycelium differs from the live mycelium in the borders of the circles in that it originates from the extension of the viable segments within the variegated hyphae (Figure 1A, 6 hours) and not from late-germinating spores, as occurs on the outer surface of the circles (see above and Discussion). The mycelium develops in the form of islands (Figure 1D, 15 hours), which appear near the edge of the circles and spread in radial waves towards the center (Figures 1D_1_-D_3_, 15 hours). This radial pattern of growth from the edge of the circles inwards leaves clear areas near the center, unoccupied by islands (Figure 1D_3_, 15 hours). Hence, at this point the plate is uniformly covered with mycelium arranged in dense circles separated by less dense areas. Figures 1D_1 _and 1D_2 _(15 hours) show a mycelial circle in Phase D; arrows indicate the edge of the circle as observed under CLSM (Figure 1D_1_) and under non-fluorescence, phase-contrast microscopy (Figure 1D_2_). The mycelium outside the circle is evident in the latter. This mycelium does not reveal fluorescence in Figure 1D_1 _at the magnification used, due to its lower density. The circles are distributed quite symmetrically (approximately one per 2.5 cm^2^), as deduced from the microscopic analyses (not shown in the pictures).

During Phase E (16–20 hours), there is profuse growth of the live mycelial islands located in the center of the circles described above. Occasionally, several eccentric circles can be seen in the center of the largest circle (Figure 1E_1_, 18 hours). Figure 1E_2 _shows a cross section of the mycelial layer during Phase E: the eccentric circles of live mycelium develop from the bottom up, forming separate layers with well defined boundaries (arrows in Figure 1E_2_). Figure 1E_3 _shows the edge of a circle during this phase. No fluorescence is observed outside the circle, owing to the lower density of mycelium located there (see also Figures 1D_1 _and 1D_2_).

Phase F (20–30 hours) is characterized by the mycelial growth between the circles described in the earlier phases (Figure 1F, 23 hours). These areas remain less dense than the circles and are made up of live hyphae.

Phase G (30–45 hours) represents the culmination of the second death round in the substrate mycelium, as well as the pre-sporulating areas of aerial mycelium [6,22-25]; Figure 1G, 38 hours]. The majority of the hyphae located in the center of the circles are dead and present segmented DNA in the nucleoids (Figure 1G_2_, 38 hours).

Phase H (45–96 hours) is the sporulation phase. Live hyphae grow and form spores inside and outside the circles (Figure 1H_1_, 80 hours). The mycelium between the circles has also suffered a second death round prior to sporulation, although it is less extensive than the one that takes place inside the circles and is not visible at the magnification shown. The change in colour (arrow in Figure 1H_1_, 80 hours) indicates the border of the original circle, which continues to reveal greater density than the areas between circles. Figure 1H_2 _(80 hours) shows a detail of the viable chains of spores.

The result of this entire process is a variability in the developmental phases across the plane of a solid agar surface. In this work will refer to this phenomenon as "longitudinal heterogeneity".

### Effect of the inoculum: density determines the presence of circles during development

**Figure 2:**
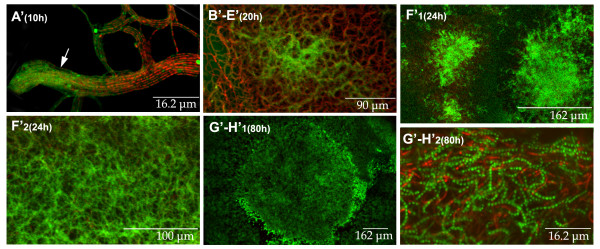
Longitudinal sections of *Streptomyces antibioticus *ATCC11891 surface cultures obtained using a diluted inoculum (6 × 10^3 ^spores per plate). The developmental phases (A'-H') and culture times are indicated. Samples were stained with SYTO 9 and propidium iodide. Arrow in picture A' indicates a group of hyphae in transition from presenting uniform green fluorescence to a variegated appearance, in which live (green) and dead (red) segments alternate in the same hypha. See text for details.

The experiments described above were performed with a sufficient density of spore inocula as to produce rapid, confluent growth on the plates and represent the conditions often encountered in the laboratory during any given morphological and physiological analysis of *Streptomyces *[26], see Methods]. When a highly diluted inoculum was used (6 × 10^3 ^spores per plate), a significant delay was observed; sporulation was less efficient, and circles did not form (Figure 2). The phases indicated with apostrophes to distinguish them from their equivalents with the undiluted inoculum) are described below.

Phase A' (0–15 hours) corresponds to the germination and first death round similar to the corresponding cycle phase obtained with a normal inoculum. The main difference encountered with respect to the normal inoculum is the higher proportion of spores that present a viable, non-variegated germ tube (Phase A; not shown). However, when density increased and the hyphae touch one another, all of them die giving rise to the variegated appearance. Figure 2A' (10 hours) shows a group of hyphae that are beginning to lose their uniform green fluorescence (arrow), developing the variegated appearance which results from the alternating live and dead segments within the same hypha (on the right of the picture). This is indicative of the change in membrane permeability revealed by the propidium iodide that stains the dying segments red (see Methods).

Phase B'-E' (15–24 hours). No late-germinating spores remain at these time points (see Discussion). The live segments of the initial mycelium begin to develop in relatively regular, fast-growing islands. In this case, a unique, exponential growth phase is observed, unlike the various growth phases seen with normal inoculum. Figure 2B'–E' (20 hours) is a superficial view of an island formed by the emerging live mycelium.

Phase F' (24–40 hours) corresponds to the growth phase of the mycelium located between the islands. It is comparable to the mycelial growth between the circles described in Phase F of the developmental cycle in the presence of normal inoculum (Figures 2F'_1 _and 2F'_2_, 24 hours)

Phase G'-H' (40–96 hours) is the sporulation phase. The mycelial distribution in islands that began in Phases B'-E' is maintained. Like the circles, the islands also delimit the areas with a higher spore density (Figure 2G'–H', 80 hours). Figure 2G'–H' (80 hours) presents a detail of the surface revealing the spore chains.

In conclusion, the main difference observed with the diluted inoculum is the absence of circles originating from the delayed spore germination. The relevance of this will be discussed later.

### Longitudinal heterogeneity determines multiphase growth curves

The methodological approach applied in this study eneabled us to obtain a high degree of synchronization in the plate cultures, thereby making it possible for us to analyze the relationships between growth rates and successive developmental steps. Figure 3 shows the growth curves (total protein and fresh weight per plate) obtained using normal or diluted inocula. The different phases described (see above) are indicated on the graph. Note the existence of three distinct stages of exponential growth in the normal cycle (indicated with arrows on the normal inoculum graph, Figure 3A), separated by two phases of temporary growth arrest (see the protein curve in Figure 3A). The second temporary growth arrest is not seen in the weight curve, probably because this parameter is less sensitive. The three waves of exponential growth correspond to Phase C (growth of the hyphae forming the edge of the circles), Phase E (rapid growth of the live hyphae from the center of the circles outwards) and Phase F (growth of the mycelium between the circles). The growth arrest periods correspond to Phase B (slow formation of the mycelial rings from late-germinating spores), Phase D (slow growth induction in the live segments of the dead hyphae located in the center of the circles) and the latter stage of Phase E (slow growth induction of the live segments of the dead hyphae located between the larger circles). Total protein per plate declines during the second death round, whereas weight continues to increase at a slower rate. This may be due to the accumulation of reserve compounds (glycogen and trehalose) within the cells [27]. Figure 3B shows the growth curves (fresh weight and total protein) for diluted inoculum (6 × 10^3 ^spores per plate). In these conditions, only two periods of exponential growth are observed, coinciding with mycelial development in the form of islands and the development of mycelium located between the islands, respectively (Figure 3B, arrows). Hence, there is a close correlation between morphological phases and growth curves. These developmental dynamics lead to the appearance of a layer of mycelium arranged in circles measuring approximately 0.5 cm or in islands measuring 0.9 mm separated by areas with a lower density of mycelium.

### The first death round and longitudinal heterogeneity are general phenomena in surface cultures of the *Streptomyces *genus

**Figure 4:**
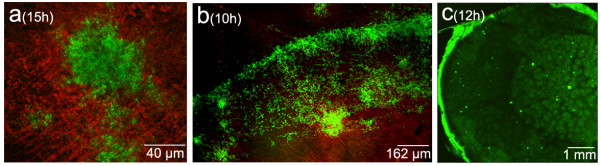
Longitudinal sections of different *Streptomyces *strains growing in confluent surface cultures. (a) *S. coelicolor *A3(2). (b) *S. glaucescens *ETH22794. (c) *S. antibioticus *ETH7451. Culture times are indicated. Samples were stained with SYTO 9 and propidium iodide.

The *Streptomyces *strains were grown in media in which the complete life cycle takes place with abundant sporulation (see Methods). All the *Streptomyces *species/strains analysed in our laboratory (with the exception of *S. coelicolor *A3(2); see below), present the pattern reported for *S. antibioticus *ATCC11891: circles when dense inocula are used and islands in the presence of highly diluted inocula. The only differences between the developmental cycles studied are the times required to reach the different phases and the diameter of the circles. Figures 4b and 4c show *Streptomyces glaucescens *ETH22794 and *Streptomyces. antibioticus *ETH7451 circles (compare with Figures 1C and 1E). As already mentioned, *S. coelicolor *A3(2) is the only exception to the described pattern: circles are not formed in any inoculum condition (concentrated or diluted) and development always occurs in the form of islands (Figure 4a).

**Figure 5:**
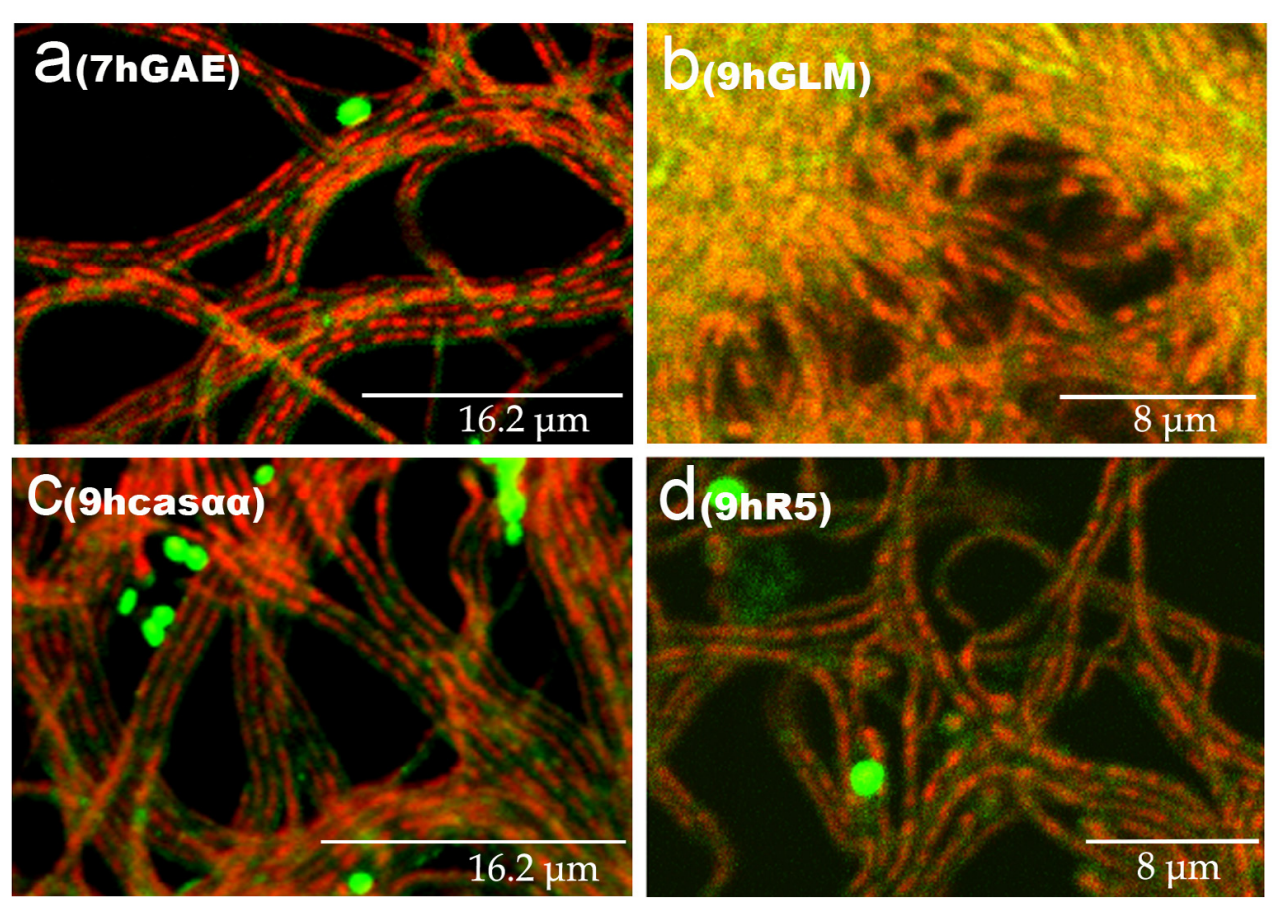
Cross sections of *S. antibioticus *ATCC11891 cultures in different solid media. Developmental time points and culture media are indicated. Casαα is GAE plus 2% casamino acids. Samples were stained with SYTO 9 and propidium iodide.

In media in which *S. antibioticus *ATCC11891 do not sporulate (GYM, GAE plus 2% casamino acids and R5; see Methods), the first death round and the variegated hyphae are also present (Figure 5) and longitudinal heterogeneity is observed (not shown). However, the second death round and sporulation are absent.

## Discussion

All studies to date describing the differentiation and developmental cycle of *Streptomyces *refer to a completely live, viable substrate mycelium that grows inside the culture medium from which a reproductive (aerial) mycelium emerges after a massive death round. The most superficial part of the aerial mycelium forms spores by septating into chains of uninucleate compartments, whereas the non-sporulating aerial mycelium and the substrate mycelium eventually die [6,22-24]. In accordance with this model, the substrate mycelium in *Streptomyces antibioticus *ATCC11891 is the mycelium formed from spores and developed until approximately 35 hours of cultivation at 28–30°C. The formation of aerial mycelium commences at this point and at approximately 60 hours of cultivation, the sporulation process begins [22,28]. The three mycelial types (substrate, aerial and sporulated aerial) exist simultaneously at certain times. Again, according to this model, this cycle is present in all *Streptomyces *species, albeit with certain temporal variations.

**Figure 6:**
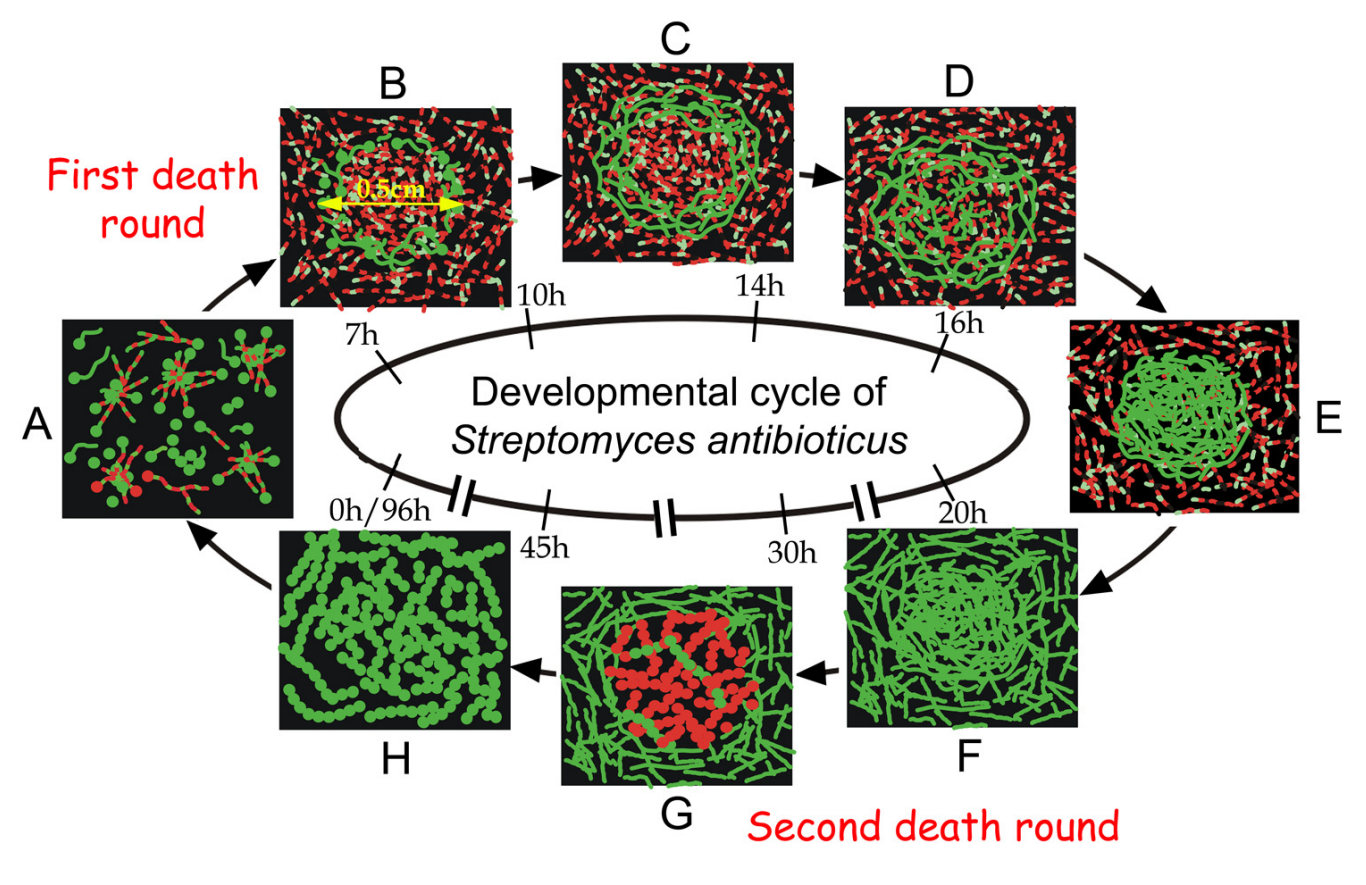
Model for the developmental cycle of *S. antibioticus *ATCC11891 on surface GAE cultures (detailed in text). Times are indicated in hours.

In this work we have analysed the developmental cycle of *Streptomyces *in three spatial dimensions, the transverse and longitudinal axes, a perspective we believe is essential to understanding the developmental cycle. The developmental features of *S. antibioticus *ATCC11891 on confluent surface cultures are summarized in Figure 6. An early death round takes place affecting the young substrate hyphae, which to date, has not been described. This death process occurs in a remarkably symmetrical form: live and dead segments alternate within the same hyphae in a very regular pattern (Figure 1A, 6 hours). Subsequently, the remaining mycelium grow in successive waves, creating longitudinal heterogeneity of the bacterium on the plate surface. In all prior studies of the *Streptomyces *developmental cycle in solid media, it is implicitly assumed that the cultures are homogeneous all over the plate surfaces; hence, the point at which the plate is analysed would not be critical. The only heterogeneity present would be in the transverse plane; that is from the bottom up. Our data clearly demonstrate that substrate and aerial mycelial growth is heterogeneous and orderly, forming a remarkable pattern of circles and islands. This finding suggests that diffusible signals are involved in their induction.

As occurs with other bacteria, *Streptomyces *cultures are niches where quorum-sensing phenomena take place [29], particularly in the relatively crowded conditions normally employed in the laboratory for growing surface cultures. Many signals of this type have been reported in the *Streptomyces *genus [30]. The absence of circles in diluted inoculum conditions might also be related to the lack of appropriate signals. The circles are formed by spores that germinate at a later time, under the influence of signals that determine their growth as viable mycelium that does not die at the earlier time points (see above). Bearing in mind that the circles initially form at very early time points (Figures 1 and 6), it can be speculated that at low inoculum densities, the hypothetical signals do not accumulate enough to induce these structures. Another simple alternative explanation concerns the origin of the circles. Given that they are initially comprised of late-germinating spores that are logically less common in highly diluted inocula, it can be hypothesized that when the mycelial layer is dense enough to produce the signals, no late-germinating spores remain and hence, the edge of the circles is not formed. The phenomena described here are clearly different from other surface phenotypes, such as the previously described *Streptomyces *"pocks" [31]. The pocks are caused by the presence of conjugative plasmids and their appearance is due to the slower rate of mycelial growth at these points [26,31]. The opposite happens with the circles described in the present work, which are made up of a high mycelial density (see above). Some authors have described the existence of circular areas of unknown origin, which were "not genuine pocks", yet were present in many *Streptomyces *cultures [26]. These areas probably correspond to the circles described in this paper.

Our data clearly show that longitudinal heterogeneity is at least as important as transverse heterogeneity in the development of *Streptomyces *surface cultures. Very different developmental phases may therefore be taking place simultaneously at two different points of the surface cultures (Figures 1 and 6). For example, in Phase E, the live mycelium inside the circles is fully developed and in the process of dying, whereas the mycelium between the circles is just beginning to grow. This is fundamental in order to properly understand and interpret the distinct stages that occur during the differentiation cycle of *Streptomyces*. With the exception of *S. coelicolor *A3(2), all the *Streptomyces *species analysed in our laboratory present the pattern reported for *S. antibioticus *ATCC11891. *S. coelicolor *do not form circles in any inoculum condition and instead, only present surface development in the form of islands (Figure 4). Knowing whether one specific species or strain of *Streptomyces *grows in "circles" or "islands" is key for the correct interpretation of the morphological/biochemical data obtained in solid cultures, as illustrated by the growth curves (Figure 3). In media in which there is no sporulation, the first death round takes place and longitudinal heterogeneity is present; however, the second death round does not occur. Consequently the first death round and the appearance of longitudinal heterogeneity are general events inherent to the development of *Streptomyces *on surface cultures. We are currently analysing the peculiarities of several *Streptomyces *species in different culture media, in order to integrate these into a consensus and create a reliable model of *Streptomyces *development on surface cultures.

A vast collection of mutant *Streptomyces *[32,33] will be generated in the future thanks to the new, powerful techniques currently available. Factoring the features described here into the analysis of these and other previously reported differentiation mutants of *Streptomyces *will greatly expand the number of potential phenotypes to be considered and hence, their corresponding genetic determinants. This in turn, will hopefully facilitate the discovery of new signal cascades in this important bacterium. Furthermore, as the differentiation of hyphae and antibiotic production share common genetic control elements [34-36], the aspects considered above will also aid in better understanding antibiotic production in solid-state fermentation [37-39], as well as in submerged conditions [40], A. Manteca and J. Sanchez, unpublished data].

## Methods

### Strains and media

*Streptomyces antibioticus *ATCC11891, *Streptomyces. antibioticus *ETH7451, *Streptomyces glaucescens *ETH22794 and *Streptomyces coelicolor *A3(2) were the species used in this research. The microorganisms were grown in solid media in which they present a complete life cycle with abundant sporulation. *S. antibioticus *ATCC11891 and *S. glaucescens *ETH22794 were grown on GAE medium [22]; *S. coelicolor *A3(2) was grown on GYM medium (glucose, yeast extract, malt extract; 41). *S. antibioticus *was also grown on media in which it does not sporulate: GAE plus 2% casamino acids, GYM [40] and R5 [25]. The cultures were prepared in Petri dishes (8.5 cm diameter) as lawns on solid medium (30 ml/plate). When indicated, sterile cellophane disks were placed on the surface prior to inoculation. Under the conditions used, the differentiation processes follow a pattern similar to that of mycelium incubated directly on the culture medium [22]. Plates (with or without cellophane) were inoculated directly with 100 μl of a spore suspension (1.5 × 10^7 ^viable spores/ml), followed by incubation at 30°C. In some cases, plates were inoculated with a highly diluted spore suspension (6 × 10^3 ^spores per plate).

### Microscopy

Culture samples were obtained and processed for microscopy at different incubation times, as described previously for submerged and surface-grown *Streptomyces *cultures [12,13]. Petri dishes prepared with Difco agar and inoculated as described above were used to obtain solid blocks of the agar cultures with a scalpel. These blocks were further trimmed to squares of approximately 10 mm in size and introduced into a hand microtome (11 mm hole diameter) previously cooled to 4°C, with the surface of growth facing sideways. Sections of about 0.3 mm were obtained (cross sections). To analyse longitudinal sections, we used solid cultures covered with cellophane disks: the disks were removed from the culture, cut into squares measuring approximately 1.5 cm × 1.5 cm and placed on slides before staining.

The permeability assay previously described for *Streptomyces *was used to stain all samples [12]. This technique involves staining the cells with cell-impermeant nucleic acid stain (propidium iodide, PI) in order to detect the dead cell population of *S. antibioticus *and with SYTO 9 green fluorescent nucleic acid stain (LIVE/DEAD Bac-Light Bacterial Viability Kit, Molecular Probes, L-13152) to detect viable cells. The SYTO 9 green fluorescent stain labels all the cells, i.e. those with intact membranes, as well as those with damaged ones. In contrast, PI penetrates only bacteria with damaged membranes, decreasing SYTO 9 stain fluorescence when both dyes are present. Thus, in the presence of both stains, bacteria with intact cell membranes appear in fluorescent green, whereas bacteria with damaged membranes appear in red [15]. The stain mixture was prepared as per the manufacturer's instructions and was added directly on the samples on the slide. The coverslide was placed on top and after staining for at least 10 minutes in the dark, the samples were then examined under a Leica TCS-SP2-AOBS confocal laser-scanning microscope at a wavelength of 488 nm and 568 nm excitation and 530 nm (green) or 630 nm (red) emission. Images were mixed using the Leica Confocal Software. In some cases, the samples were also examined in differential interference contrast mode, available with the same equipment.

### *Streptomyces *cell sampling and processing

Cells from *S. antibioticus *grown on the surface of cellophane disks were scraped off with a plain spatula at different time points. Growth curves were obtained by means of weight determinations and total protein analysis. Fresh weight data (obtained without subjecting the mycelium to any drying treatment) were used, given that these data are reproducible and afford reliable determination of growth values in the early stages of the cultures when the small amount of mycelium per plate at these time points makes the use of other alternative approaches difficult. Determinations were repeated a minimum of three times. Total protein was analysed in the collected mycelium as follows: the weighted mycelium was resuspended in buffer A (Tris-HCl 20 mM pH 8, EDTA 1 mM, β-mercaptoetanol 7 mM and PMSF 0.5 mM), maintaining a constant ratio between fresh weight of mycelium and volume of buffer A (65 mg mycelium/ml buffer A). The suspension was ruptured in an MSE soniprep 150, in 6 cycles of 10 seconds, on ice, after which the samples were centrifuged at 10000 r.p.m. in an Eppendorf microcentrifuge for 30 min at 4°C and the protein was determined by the Lowry assay [42].

## List of abbreviations

CLSM, confocal laser-scanning fluorescence microscopy; PI, propidium iodide.

## Authors' contributions

AM performed all microscopic and biochemical analyses. MF participated in some of the experiments and in the initial study design. JS conceived the study, participated in its design, and coordinated and drafted the manuscript. All authors read and approved the final manuscript.
